# Therapeutical Notes

**Published:** 1900-05-12

**Authors:** 


### THERAPEUTICAL NOTES.

### Hæmoptysis.

Scarpa uses the following formula for the arrest of bleeding from the lungs. If there is much cough he adds morphine or codeia :—

℞. Ext. fluidi Hydrastis Canadiensis.
Tinct. Hydras. Canad. āā min. 225.
Misce. S. min. 20 to min. 50.

To be taken three times a day. The treatment to be continued two or three days after the bleeding has ceased.—*New York Med. Jour.*, November 25th.

### Earache.

Richards uses glycerine bougies made in a urethral bougie mould for early stages of otitis media. The affected ear is placed uppermost, and the bougie, cut to the length of the meatus, is allowed to slip down into it. They contain carbolic acid 3½ per cent., fluid extract of opium 3 per cent., cocaine and sulphate of atropine of each 1½ per cent. Not only is pain relieved, but the glycerine draws out some serum and lessens the tension, so that paracentesis may be obviated in some instances. —*Practitioner*, December.

### Chloretone, the New Hypnotic.

This substance is said to produce sleep without affecting the heart or tension of the blood-vessels, and it has, therefore, been recommended in cardiac and senile cases. Moreover, it possesses definite powers in relieving pain, and it can be used as a local application to wounds, burns, and as an anæsthetic for small operations in place of cocaine. Vomiting is stopped by small doses taken internally. For causing sleep 6 to 20 grains are taken in a single dose, followed by a draught of milk or water. It appears to be broken up in the body, for no traces of it can be found in the excreta.—*Jour. Amer. Med. Assoc.*, p. 777, 1899.

### A Painless Blister.

A very good formula for a vesicant plaster, the use of which is not attended by pain, is

Menthol.

Chloral āā, ʒi.

Spermaceti, ʒiv.

Ol. theobrom, ʒii. Misce.

Spread on a piece of linen.—*The Medical Press*, February 7th.

